# Molecular Mechanisms of RADA16-1 Peptide on Fast Stop Bleeding in Rat Models

**DOI:** 10.3390/ijms131115279

**Published:** 2012-11-19

**Authors:** Ting Wang, Xiaozhong Zhong, Songtao Wang, Fei Lv, Xiaojun Zhao

**Affiliations:** 1 Institute for NanoBiomedical Technology and Membrane Biology, West China Hospital, Sichuan University, Chengdu 610041, Sichuan, China; E-Mails: ting.wang1019@gmail.com (T.W.); april-2005@163.com (S.W.); lvfei0424@sina.cn (F.L.); 2 College of Life Science, Jianghan University, Wuhan 430056, Hubei, China; E-Mail: saisky112@163.com; 3 National Engineering Research Center for solid state fermentation, Luzhou 640040, Sichuan, China

**Keywords:** RADA16-1, nanofiber scaffolds, self-assembly, hemostasis

## Abstract

Ionic self-assembly of the peptide RADARADARADARADA (RADA16-1) may form a well-defined nanofiber and eventually a hydrogel scaffold, with a water content of over 99.5%. This leads to the establishment of a nanofiber barrier that can be used to achieve complete hemostasis in less than 20 s in multiple tissues and in a variety of different wounds. In the present study, the nanofiber scaffolds of RADA16-1 peptide were sonicated into smaller fragments to identify possible molecular mechanisms underlying the rapid cessation of bleeding associated with these materials. Atomic force microscopy (AFM), circular dichroism (CD), and rheometry were also used to evaluate the re-assembly kinetics of this peptide. A bleeding control experiment was performed in animal models to uncover the molecular mechanisms underlying this fast hemostasis. In this way, these sonicated fragments not only quickly reassembled into nanofibers indistinguishable from the original material, but the degree of reassembly was also correlated with an increase in the rigidity of the scaffold and increased as the time required for hemostasis increased.

## 1. Introduction

Previous studies on utilizing self-assembling peptides have shown that they can be used to establish nanofiber barriers that, when applied directly to a wound in the brain, spinal cord, femoral artery, liver, or skin of a mammal, could achieve complete hemostasis in less than 20 s [1]. This therapy stops bleeding without the use of pressure, cauterization, vasoconstriction, coagulation, or cross-linked adhesives [2–5]. Unlike other hemostatic agents, self-assembling peptides do not have any pyrogenicity and do not undergo systemic coagulation, which can be a safety issue in animals [6]. This reduces the risk of secondary damage and of the problems caused by the chemical and biological contaminants that are typically present in animal-derived biomaterials such as collagen.

**Figure 1:**
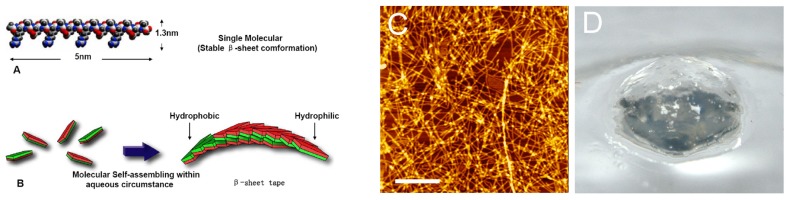
Peptide RADA16-1. (**A**) Molecular model of the RADA16-1; (**B**) Molecular model of numerous RADA16-1 as they undergo self-assembly to form nanofibers with the hydrophobic alanine sandwiched inside and hydrophilic residues on the outside; (**C**) The RADA16-1 nanofibers was examined by using atomic force microscopy (AFM); (**D**) RADA16-1 gelatinized into hydrogel.

RADA16-1 was a member of the designed self-assembling peptides family. The primary sequence was [COCH3]-RADARADARADARADA-[CONH2], The regular repeats charged residues within the primary sequences and its extremely stable β-sheet conformation in water make them organize complementary ionic bonds on the hydrophilic surface. The monomer of RADA16-1 was ≈5 nm long, ≈1.3 nm wide, and ≈0.8 nm thick in dimensions [7–9] (Figure 1A). RADA16-1 samples can spontaneously assemble nanofibers ranging from a few hundred nanometers to a few microns in length (Figure 1B,C); furthermore, the self-assemble action not only forms stable nano scale fibers, but also forms higher-order interweave nanofiber scaffolds: namely, hydrogels with extremely high water content [≥99.5 (*w*/*v*)% water], by changing to neutral pH or adding physiological concentrations of salt solutions *i.e.*, saline, physiological solutions, blood or cerebrospinal fluid [10–12]. The hydrogel peptides formed were achromatic color, hyaloid and can be fabricated into various geometric shapes (Figure 1D).

Nanofiber structures of some self-assembling peptides have been studied extensively, but the molecular mechanisms underlying self-assembly and reassembly, which are correlated with fast bleeding control, still remain unclear [13]. We asked whether the dynamic self-assembled nanofibers could influence the hemostatic process. In this study, RADA16-1 was sonicated into smaller fragments. Atomic force microscopy (AFM), circular dichroism, and rheology were used to determine the kinetics of re-assembly. A bleeding control experiment was performed in animal models to identify possible molecular mechanisms underlying this hemostasis. We report here that the sonicated fragments of RADA16-1 can quickly reassemble into nanofibers indistinguishable from the original material and that they can also self-assemble into long fibers. The disassembly and re-assembly processes were repeated three times. The reassembled nanofibers had the same length each time. The hemostatic process showed a relationship to the length of the self-assembled nanofibers. The longer the nanofibers, the higher the storage modulus G′ of the RADA16-1 hydrogels, and the shorter the duration of bleeding. Our findings not only facilitate further development of novel surgical hemostasis systems but may also have significance for the study and fabrication of a broad range of fibrous biological materials.

## 2. Results

**Figure 2:**
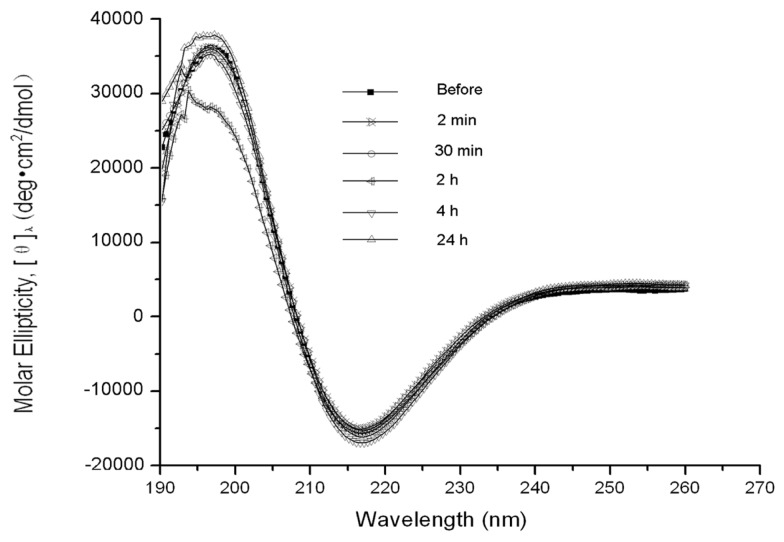
CD examination of the peptide structures at various times before and after sonication: 2 min, 30 min, 2 h, 4 h, and 24 h. Typical β-sheet spectra were observed, indicating that molecular structure and the integrity of the peptides were the same before and after sonication. The concentration of β-sheets (216 nm) was slightly higher after sonication than before, suggesting tight β-sheet packing.

The β-sheet structure of the backbone was essential to RADA16-1 self-assembly. CD examination of the peptide structures was used to measure the concentration of β-sheets at various times before and after sonication (Figure 2). Typical β-sheet spectra were observed at each point in time, indicating the molecular structure and the integrity of the peptides was the same before and after sonication. The concentration of β-sheets (216 nm) was higher after sonication than before, suggesting tight β-sheet packing.

**Figure 3:**
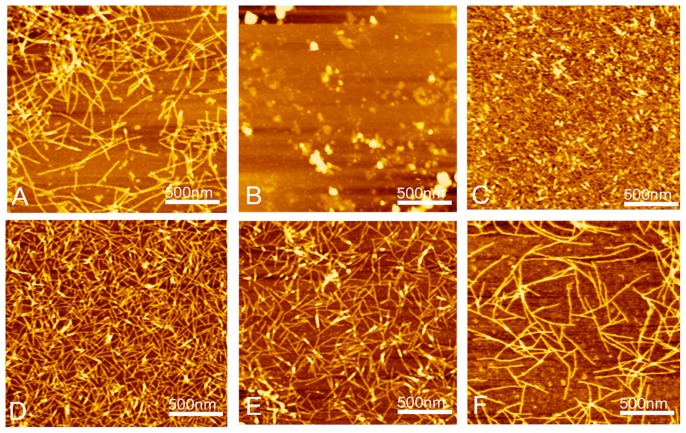
AFM images of RADA16-I nanofibers at various points in time: (**A**) before sonication; (**B**) 2 min after sonication; (**C**) 30 min; (**D**) 2 h; (**E**) 4 h; and (**F**) 24 h. Note the elongation and reassembly of the peptide nanofibers over time.

**Figure 4:**
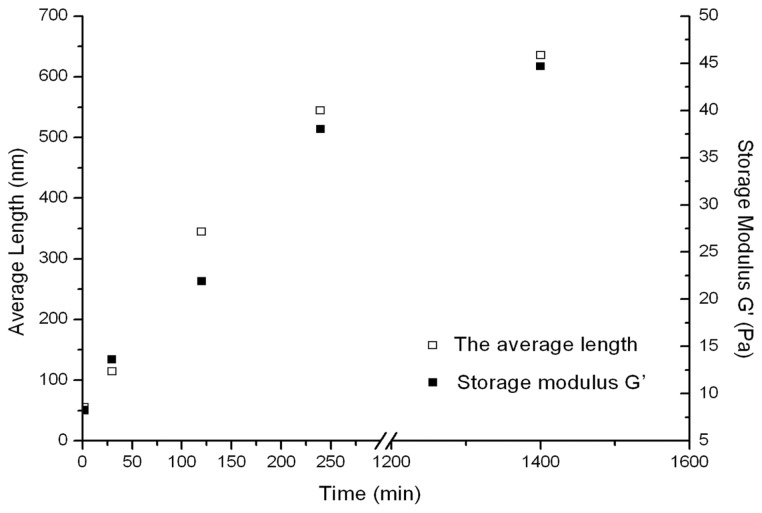
Kinetics of the reassembled length of RADA16-I nanofibers obtained from AFM and increase in the elasticity of RADA16-I scaffold hydrogel after sonication followed by rheometry. Storage modulus G′ (Back square) is shown as a function of time (right *y* axis) and of the average length (red square) of the RADA16-I fibers (left *y* axis).

AFM clearly showed that RADA16-1 forms a long self-assembled nanofibers ranging with hundred nanometers, about 615.4 ± 103.54 nm (Figure 3A,F). To assess the structural stability of the nanofibers, the RADA16-1 hydrogel solution was sonicated for 1 min to break the fibers. AFM analysis showed that most of the RADA16-1 nanofibers were broken into small fragments with an average length of about 21.22 ± 5.45 nm (Figures 3B and 4), 2 min after sonication. These small fragments retained the ability to self-assemble and finally reassembled into long nanofibers with an average length of about 615.4 ± 103.54 nm 1440 min after sonication (Figures 3F and 4). All the above experiments were repeated three times. Results showed that the small fragments can self-assemble into nanofibers in a stable and repeatable manner.

The rheology of reassembly as a function of time is shown in Figure 4. The reassembled peptide nanofibers were subjected to sonication three additional times. In each case, the hydrogels were found to reach the storage modulus G′ from about 6 Pa at 2 min after sonication to about 36.5 Pa at 240 min after sonication. At the finial checkpoint (1440 min after sonication) the storage modulus G′ (Pa) of the scaffold hydrogels reached about 50 Pa, which is indistinguishable from that of unsonicated materials.

**Figure 5:**
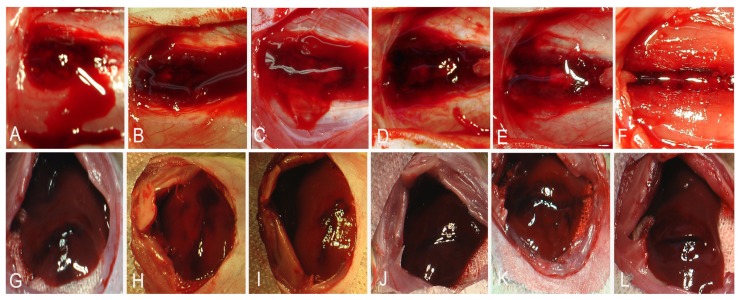
Hemostasis at (**A**–**F**) spinal cord transection and (**G**–**L**) rat liver transverse cut. RADA16-1 solution was applied at various points in time before and after sonication. The time required for complete cessation of bleeding was recorded: (**A**,**G**) 2 min, (**B**,**H**) 30 min, (**C**,**I**) 120 min, (**D**,**J**) 240 min, (**E**,**K**) 1440 min, and (**F**,**L**) pre-sonication. Note the profuse bleeding.

**Figure 6:**
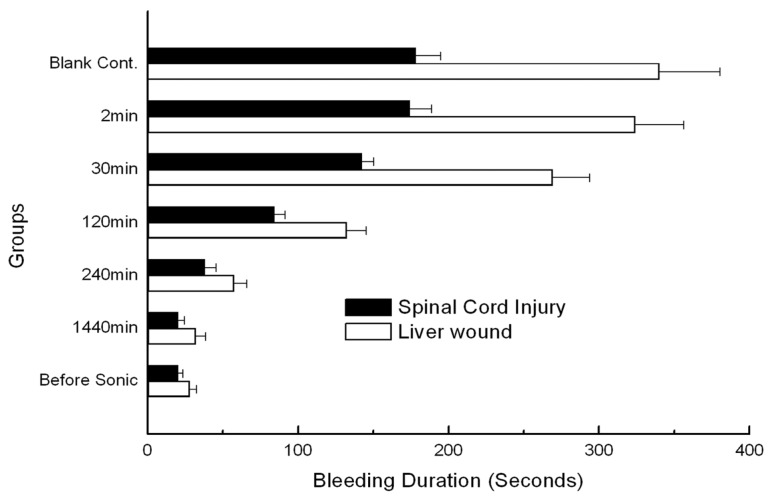
Time required for complete hemostasis. Graphs show the duration of bleeding in cases treated with 1% RADA16-1 self-assembling peptide solution relative to saline-treated samples (blank control group) in the case of spinal cord transection and liver sagittal cuts.

After laminectomy and removal of the dura, the spinal cord was transected at T8, from the dorsal to ventral aspect (Figure 5A–F). Each cord was then and treated (*n* = 5) with 50 μL of 1% RADA16-1. Hemostasis was achieved within 20 s in the pre-sonication groups and 1440 min post-sonication groups (Figure 6). In the saline controls (*n* = 5) and 2 min post-sonication groups (*n* = 5) bleeding continued for over 3 min. Each treated group was compared to the saline controls and significant differences were identified using the Tukey test with a 95% confidence interval.

In the liver experiment, a sagittal cut was made in the left lobe (Figure 5G–I). Upon treatment with 200 μL of 1% RADA16-1 solution bleeding ceased in less than 20 s (Figure 6). In the saline controls (*n* = 5) and groups cut 2 min after sonication, bleeding continued for more than 300 s. Each test group was compared to saline-treated controls and significant differences were identified using the Tukey test with a 95% confidence interval.

**Figure 7:**
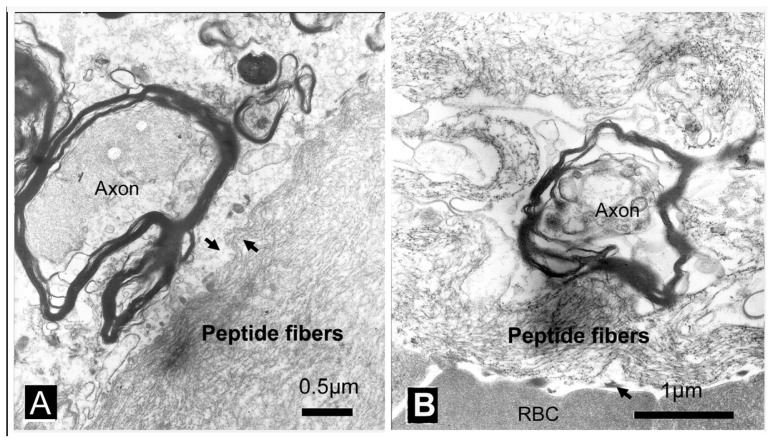
Transmission electron microscopy (TEM) images showing the RADA16-1 nanofibers and interactions between RADA16-1 with tissues: (**A**) The cut in the spinal cord was treated with RADA16-1. Note the tight interactions between the irregular interfaces (black arrow). (**B**) A tiny vacant space presented between red blood cells (RBCs) and RADA16-1 nanofiber scaffolds at the interface (black arrow). Scale bar represents A = 0.5 μm, B = 1 μm.

The treated tissue was examined using transmission electron microscopy (TEM) to determine the molecular mechanism and the relationship between the RADA16-1 and the blood/tissue interface. 1% RADA16-1 was applied to a spinal cord lesion and the tissue was harvested at 30 min after injury. In the electron micrograph, there was no evidence of platelet aggregation at the blood/RADA16-1 nanofiber interface. A very tight interaction was found between RADA16-1 nanofibers and the neural tissue (Figure 7A). A tiny space presented between red blood cells (RBCs) and RADA16-1 nanofiber scaffolds at the interface (Figure 7B). RBCs were removed from the lesion by RADA16-1 but there was no evidence of lysing.

## 3. Discussion

The peptide RADA16-1 contains alternating hydrophobic and hydrophilic residues. Hydrophilic residues contain alternating negative and positive charges (Figure 1A) [14]. This type of peptide can form β-sheets structure and self-assemble into the nanofibers and eventually hydrogels consisting of >99.5% water (Figure 1B–D). CD analysis has confirmed that RADA 16 assumes a β-sheet structure with minimal ellipticity at 217–218 nm, maximal ellipticity in the 195 nm region, and an isosbestic point at 207 nm (Figure 2). CD analysis showed sonication to be unable to disrupt the β-sheet structure (2D) and showed small fragments to quickly self-assemble into nanofibers. AFM clearly showed that most of the RADA16-1 nanofibers are broken into small fragments with an average length of 21.22 ± 5.45 nm (Figure 3A), 2 min after sonication. These small fragments retained their ability to self-assemble and formed long nanofibers with an average length of 543.47 ± 67.42 nm 240 min after sonication (Figure 3E). They eventually formed nanofibers of up to 615.4 ± 103.54 nm 1440 min after sonication (Figure 3F). These fibers were indistinguishable from the original material (Figure 3A). In a rheology experiment, the viscoelastic property increased as a function of time and the length of the self-assembled nanofibers. The storage modulus G′ (Pa) of the scaffold hydrogels became higher, eventually reaching ≈50 Pa, but the nanofibers reassembled into ≈600 nm fibers on average (Figure 4). These observations were consistent across three additional tests. The longer the nanofibers, the higher the storage modulus G′.

The results of these experiments provide several clues, which may contribute to the hemostasis. First, hemostasis could not be explained by clotting. No blood clots were observed during the experiments (Figure 5). The electron micrographs showed no evidence of platelet aggregation at the interface between the nanofiber barriers and wound site (Figure 7A) [15–17]. Second, As solution of 1% RAD16-1 was about pH 3.5, and extracellular matrix has positive charge, it was understandable that through electrostatic interaction, peptide fibers absorbed on the edge of wound and contributes as anchor as fasten hydrogel to seal the wound. Third, in the experiments, RADA16-1 was highly hydrated, showing about 99% water content. It filled an irregular void before assembly and then assembled to form the molecular nanofiber scaffolds. This *in situ* self-assembly property may be critical because most other materials do not conform or form tight contacts in the irregular voids created by injuries. Few other materials can drive RBCs out of the lesions the way RADA16-1 can, as shown in the electron micrographs (Figure 7B).

Hemostasis can be explained using gelatinization kinetics. Applying the RADA16-1 solution to the wounds 2 min post-sonication did not arrest bleeding much more than application at 3 min. Both were similar to blank controls. The hydrogels fractured at the tissue interface. Within the resultant hydrogels, the lesions bled profusely. This may have been caused by the shortness of the nanofiber and by the low viscoelasticity of hydrogels (Figure 5A,G). As the storage modulus G′ became higher, the time required to control bleeding decreased, eventually within about 20 s. The hydrogels flexed with the pulse. The storage modulus G′ (Pa) of the scaffold hydrogels, shown as a function of time, increased as the length of the self-assembling nanofibers increased (Figure 4).

## 4. Experimental Procedures

### 4.1. Materials

The peptides used in this study (RADA16-I, [COCH_3_]-RADARADARADARADA-[CONH_2_]) were synthesized from Chengdu CP Biochem L.T.D. and stored at −20 °C until needed. The *N*-terminus and *C*-terminus were protected by acetyl and amino groups (molecular weight 1712 Da, purity 95%). Peptides were synthesized using standard solid-phase chemistry. Peptide homogeneity and composition were analyzed by analytical HPLC and mass spectrometry. Stock solutions of the peptides were prepared at concentrations of 10 mg/mL (1%) in Milli-Q water (18.2 MΩ, Millipore Milli-Q system, Boston, MA, USA) and stored at 4 °C. The peptide solution was sonicated for 30 min using an ultrasonic cleaner (50 T, VWR Scientific, West Chester, PA, USA) before measurement at the maximum power setting.

### 4.2. Circular Dichroism (CD) Spectroscopy

CD data were gathered at 25 °C on an AVIV model 400 spectrometer (AVIV Associates, Lakewood, NJ, USA), using a 1 mm path length quartz cuvette. Spectra were collected at 2 nm intervals and band widths of 2 nm from 190 to 260 nm at 3 s signal intervals, on average. The baseline CD signals of the empty cuvette and baseline spectrum of a cuvette containing only pure water under identical conditions were checked. All spectra were corrected by subtracting the baseline and the data are expressed as mean residue ellipticity, [θ], which is given in (deg·cm^2^·dmol^−1^). Peptide from stock solutions (1%) was diluted to different concentrations with sodium chloride solutions. The final concentrations of the peptide solutions for CD measurement were 0.23 mg/mL (200 μM). The samples of the sonicated peptides were removed 2, 30, 120, 240, and 1440 min after sonication. Samples removed before sonication served as controls.

### 4.3. Atomic Force Microscopy

AFM was used to determine the nanostructure of the peptide. Aliquots of 5 μL peptide solution were evenly deposited onto a freshly cleaved mica surface. Each sample was left on the mica surface for about 20 s. The surface was then rinsed with 200 μL of Milli-Q water to remove unattached peptide. The samples were covered with Petri dishes to prevent contamination and then air-dried for AFM observation. AFM was performed at room temperature using the tapping mode on a SPI4000 Probe Station and SPA-400 SPM Unit (Seiko Instruments Inc., Chiba, Japan). All images utilized a 20 μm scanner (400), an Olympus Si-DF20 cantilever (Olympus Corp., Tokyo, Japan), and a 10 nm Si tip, a 12.00 N/m spring constant with a resonance frequency of 127.00 kHz. All of the measurements were performed in ambient air. Height images were recorded at a pixel resolution of 512 × 512 pixels. For each sample, images were scanned and collected at scales of 5 × 5 μm^2^, 2 × 2 μm^2^, and 1 × 1 μm^2^. The images at scale of 2 × 2 μm^2^ were displayed to show the details of the nanostructures.

### 4.4. Rheology Properties

Rheology experiments on the RADA16-1 hydrogels were performed at 25 °C on a rheometer (AR2000, TA Instruments, New Castle, DE, USA) with a cone and plate geometry system (cone diameter: 2 cm, angle: 1°, truncation: 46 μm). After sonicating RADA16-I solution for 30 min, 500 μL of the solution was placed on the plate, and the cone was lowered and eventually the tip was 25 μm above the plate. Storage modulus G′ was measured at a constant frequency of 1 Hz. The sample was tested with 2 μm oscillatory torque at 25 °C.

### 4.5. Animals and Experimental Groups

Adult Sprague-Dawley rats were anesthetized with an intraperitoneal injection of ketamine (50 mg/kg). The rats weighed between 200 and 250 g. They were randomly divided into 6 groups as follows: (1) Pre-sonication group (*n* = 5); (2) 2 min after sonication group (*n* = 5); (3) 30 min after sonication group (*n* = 5); (4) 2 h after sonication group (*n* = 5); (5) 4 h after sonication group (*n* = 5), and (6) 24 h after sonication group (*n* = 5). All animals were purchased from the Center of Laboratory Animals of Clinical Medicine School of Sichuan University. Surgical procedures were also performed at the center. All procedures were consistent with guidelines issued by the animal care and use committee of Sichuan University.

### 4.6. Spinal Cord Transection Experiment

Under an operating microscope, the eighth thoracic spinal cord segment (T8) was identified and then a dorsal laminectomy was performed in anesthetized adult rats. After opening the dura mater, we performed a transection using standard angle blades. Immediately after the cord transection, 50 μL 1% RADA16-1 was applied to the cut area at various times before and after sonication to control bleeding. The blank controls received a saline treatment. The animals were allowed to survive for as long as 4 months as part of another experiment.

Bleeding was stopped in the following manner: After making the hemorrhage wound on the experimental animal, the duration of bleeding was recorded and the material solution was injected at the moment of complete hemostasis. The area was observed for 30 s. If no new blood exuded through wound, the cessation of bleeding was considered successful.

### 4.7. Liver Transverse Cut Experiments

Rats were anesthetized and placed on their backs. Their abdomens were opened, exposing the liver. The left lobe of the liver was cut transversely using a No.15th scalpel. Before and after sonication, 200 μL 1% RADA16-1 was applied to the site of injury. The livers of control rats were treated with saline.

### 4.8. Transmission Electron Microscopy

The rats were anesthetized by overdose and perfused via a right ventricle with 100 mL NS, followed by 200 mL of fixative (4% paraformaldehyde in 0.1 M phosphate buffer (pH 7.4). The whole spinal cords of rats were fixed. Upon completion of fixation, a segment of spinal cord about 0.4 cm in length encompassing the lesion site was acquired. The specimens were fixed in 2.5% glutaraldehyde or 4% paraformaldehyde in phosphate buffer (pH 7.4) for overnight, washed in phosphate buffer (pH 7.4), pre-fixed in 1% osmium tetroxide in phosphate buffer (pH 7.4), and dehydrated in increasing concentrations of alcohol from 60% to 100%. The tissues were then washed with propylene oxide and embedded in epoxy resin embedding media. Semi-thin sections about 0.5 μm in thickness were stained with toluidine blue and examined under a light microscope. Ultrathin sections 70 nm in thickness were cut with a glass knife on an ultramicrotome. Semithin sections were stained with methylene blue. Ultrathin sections were collected on a copper grid, stained with uranyl acetate and lead citrate, and examined under a Hitachi H600 transmission electron microscope (Hitachi, Tokyo, Japan).

## 5. Conclusions

This investigation demonstrated that the self-assembling peptide RAD16-1 (1%) has a reproducible, dynamic reassembly process that produces well-ordered peptide nanofiber scaffolds. A RADA16-1 concentration correlated with reliable hemostatic efficacy under normal clotting conditions. Because the peptide can dissolve in water, it is convenient to store, transport, and use. It can be chemically synthesized and degrades naturally over time in tissues, producing only amino acids. It is naturally free of pathogens, viruses, and contaminants, which is highly relevant to clinical use [18–20]. Previous studies have shown that self-assembling peptides can support the attachment of a variety of mammalian primary cells in tissue culture [21–26]. These characteristics offer an additional benefit for wound healing after hemostasis [27,28]. The supramolecular self-assembly and reassembly event we uncovered here is likely to be widespread in many unrelated fibrous biological materials [29–31]. Self-assembly and reassembly are very important properties for the fabrication of novel materials [32–35]. A full understanding of the processes underlying self-assembly is necessary for the design of better biological materials.
